# Homeobox A5 activates p53 pathway to inhibit proliferation and promote apoptosis of adrenocortical carcinoma cells by inducing Aldo-Keto reductase family 1 member B10 expression

**DOI:** 10.1080/21655979.2021.1924545

**Published:** 2021-05-23

**Authors:** Danyan Chen, Zhaonan Shen, Xi Cheng, Qi Wang, Junlin Zhou, Fang Ren, Yue Sun, Hongman Wang, Rongxi Huang

**Affiliations:** a Departments of Endocrinology, Chongqing General Hospital, University of Chinese Academy of Sciences, Chongqing, China; b Departments of Nephrology, The Fifth People’s Hospital of Chongqing, Chongqing China; c Departments of Science & Education, Chongqing General Hospital, University of Chinese Academy of Sciences, Chongqing, China; d Departments of Laboratory, Chengdu Sixth People’s Hospital, Chengdu, Sichuan Province China; e Departments of Endocrinology, The First Affiliated Hospital of University of South China, Hengyang, Hunan Province China; f Departments of Emergency, Chongqing General Hospital, University of Chinese Academy of Sciences, Chongqing China; g Departments of Geriatrics, The First Affiliated Hospital of Chongqing Medical University, Chongqing China

**Keywords:** Adrenocortical carcinoma, Aldo-Keto reductase family 1 member b10, hoxa5 protein, human, hyperaldosteronism

## Abstract

Aldo-Keto Reductase Family 1 Member B10 (AKR1B10) and Homeobox A5 (HOXA5) are both down-regulated in adrenocortical carcinoma (ACC), and HOXA5 is predicted to bind to the promoter of AKR1B10. We aimed to investigate whether HOXA5 could bind to AKR1B10 to regulate ACC cells proliferation and apoptosis. The expression of AKR1B10 and HOXA5 in ACC patients and the relationship of their expression between ACC prognosis were evaluated by searching database. Then, NCI-H295R cells were overexpressed to detect the alteration of cell proliferation, apoptosis and the expression of p53 and p21 proteins. The interaction between AKR1B10 and HOXA5 was validated by luciferase report and chromatin immunoprecipitation. Finally, NCI-H295R cells were silenced with HOXA5 in the presence of AKR1B10 overexpression, and then cell proliferation and apoptosis were also assessed. Results revealed that AKR1B10 and HOXA5 are down-regulated in ACC patients and the low expression of it is correlated with low percent of overall survival (OS) and disease free survival (DFS). Compared with Y1 cells, SW-13 and NCI-H295R cells exerted lower expression of AKR1B10 and HOXA5. AKR1B10 significantly inhibited cell viability, colony formation and expression of Ki67 and PCNA, but promoted apoptosis and expression of p53 and p21 in NCI-H295R cells. HOXA5 could interact with AKR1B10 and enhance AKR1B10 expression. Furthermore, HOXA5 knockdown obviously blocked the effect of AKR1B10 overexpression on NCI-H295R cells proliferation and apoptosis. In conclusion, HOXA5 could bind to AKR1B10 promotor to increase its expression, activate p53 signaling, thereby inhibiting proliferation and promoting apoptosis of ACC cells.

## GRAPHICAL ABSTRACT

**Figure:**
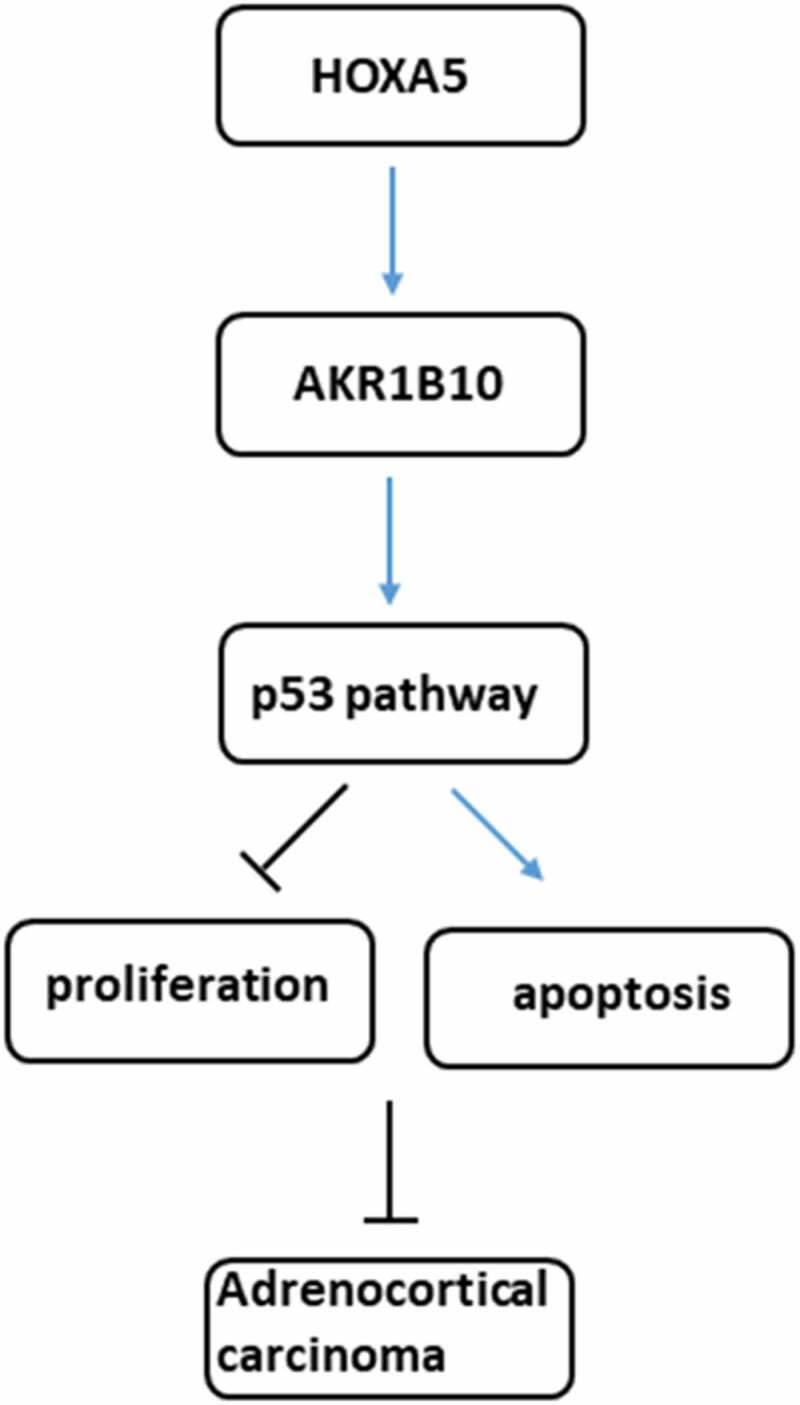

## Introduction

Adrenocortical carcinoma (ACC), a rare endocrine aggressive malignancy, occurs in the globular zone of the adrenal cortex which can secrete aldosterone [1,2]. The occurrence rate of ACC ranges from approximately 1 to 2 cases per million persons per year [3]. ACC carries a poor prognosis due to its tendency to metastasize before diagnosis and has a high risk of relapse post radical surgery [4]. Therefore, it is of great clinical significance to screen the differentially expressed genes of ACC and to explore the molecular mechanism of this disease.

Aldo-Keto Reductase Family 1 Member B10 (AKR1B10) is an oxidoreductase with NADPH (reduced nicotinamide adenine dinucleotide phosphate) as a coenzyme, and can catalyze the conversion of aldehydes and ketones into corresponding alcohols [5]. Studies have found that AKR1B10 is highly expressed in liver cancer, breast cancer, lung cancer and other tumor tissues [6–8]. Nevertheless, it is reported that compared with adjacent normal colorectal tissues, AKR1B10 in colorectal cancer (CRC) tissue is down-regulated and is related to the patient’s clinic pathological conditions. Loss of AKR1B10 promotes the proliferation and migration of CRC cells in vitro [9]. In addition, the results in the Gene Expression Profiling Interactive Analysis (GEPIA) database also shows that the expression of AKR1B10 is significantly down-regulated in the tissues of patients with ACC, and is related to the poor survival of patients. However, whether AKR1B10 was down-regulated in ACC cells and played a role in the progression of ACC has not been illustrated.

Homeobox A5 (HOXA5) is a member of the HOX family, and its encoded protein is a transcription factor that is widely involved in the normal physiological and pathological processes of the human body via regulating human embryonic development and adult stem cell differentiation [10]. The role of HOXA5 in promoting or suppressing cancer in malignant tumors from different tissues has been gradually clarified. For example, the loss of HOXA5 in breast cancer cells can promote tumor cells to differentiate in the direction of cancer stem cells, thereby promoting tumor progression [11]. In CRC, HOXA5 is down-regulated and its expression induces loss of the cancer stem cell phenotype, preventing tumor progression and metastasis by inhibiting wnt signaling [12]. Moreover, HOXA5 has been reported to cooperate with p53 to play a role in lung cancer mammary tumorigenesis [13,14]. Notably, HOXA5 is also down-regulated in ACC and it is predicted to bind to the promoter of AKR1B10 after searching JASPAR database. Therefore, we speculated that HOXA5 could bind to the promoter of AKR1B10 to modulate ACC progression.

In the present study, we aimed to investigate the expression of HOXA5 and AKR1B10 in ACC cell lines and to clarify whether HOXA5 could bind to the promoter of AKR1B10 and regulate its expression, thereby modulating ACC cells proliferation and apoptosis through p53 signaling. Our findings might provide a novel therapeutic target for the treatment of ACC.

## Materials and methods

### Bioinformatics analysis

GEPIA is a time-saving and intuition web application that is used for gene expression analysis based on abundant data from TCGA and the GTEx databases. AKR1B10 and HOXA5 mRNA expression in ACC tissues and normal adrenocortical samples was obtained with GEPIA. Additionally, the survival analysis with AKR1B10 or HOXA5 subgroup and the relationship between AKR1B10 or HOXA5 and clinicopathological information were also analyzed.

### Cell culture and transfection

Human adrenal normal cell line (Y1) and ACC cell lines (SW-13, NCI-H295R) were obtained from ATCC (Manassas, USA) and cultured in DMEM (Gibco, USA) containing 10% fetal bovine serum (FBS) and 100 U/mL penicillin-100 μg/mL streptomycin (Beyotime, Shanghai, China) in a 5% CO_2_ incubator at 37°C.

Full-length cDNAs of human AKR1B10 (overexpression-AKR1B10, Oe-AKR1B10) or HOXA5 (Oe-HOXA5) were cloned into the pcDNA3.1 vector (Thermo Fisher Scientific, Inc.). A pcDNA3.1 empty vector was used as a negative control (Oe-NC). The short hairpin RNA (shRNA) against HOXA5 (shRNA-HOXA5) and negative control shRNA (shRNA-NC) were designed and synthesized by GeneScript (Nanjing, China). NCI-H295R cells in logarithmic growth phase were selected and seeded in the 6-well plates. Cell transfection was performed when the cell confluence was up to 50%-60% according to the instructions of Lipofectamine 2000 (Invitrogen, USA). Following 48 h of transfection, the cells were collected for subsequent experiments.

### Cell counting kit-8 (CCK-8)

NCI-H295R cells that transfected with indicated vectors were cultured in 96-well plates with 2 × 10^3^ per well. Subsequently, 10 µl CCK-8 reagent (Beyotime, China) was added to each well after cell culture for 24, 48 and 72 h, and incubated at 37°C for 2 h in the dark. The optical density of each well was measured at a wavelength of 450 nm using a microplate reader (Bio-Rad, USA).

### Colony formation assay

NCI-H295R cells in the logarithmic growth phase were seeded for colony formation assay into dishes at a density of 200 cells per dish. The cell culture medium was replaced every 3 days and cell culture was terminated when macroscopic colonies could be observed. Colonies were then washed with PBS, fixed with 4% paraformaldehyde and stained with Gimsa solution for 10–30 min. The number of colonies with over 10 cells were photographed with a microscope at low magnification (×100).

### Western blot

Total proteins from cells were extracted using RIPA lysis buffer (Beyotime Biotechnology, China) containing protease inhibitor (Roche, Switzerland). Protein samples were subjected to SDS-PAGE and then transferred onto PVDF membranes (Roche, Switzerland). After being blocked with 5% nonfat milk for 2 h at room temperature, the membranes were incubated with primary antibodies (Abcam, UK; 1:1000 dilution) against AKR1B10, Ki67, PCNA, Bcl-2, Bax, cleaved-caspase3 (c-caspase3), pro-caspase3, c-caspase9, pro-caspase9, p53, p21, HOXA5 and GAPDH at 4°C overnight. The membranes were then being incubated with a goat anti-mouse/rabbit secondary antibody (Abcam, UK) for 2 h at room temperature. Enhanced chemiluminescence reagent (Thermo Fisher Scientific, Inc., USA) was used to visualize the protein bands in a Bio-Rad ChemiDoc XRS Imaging system (Hercules, USA).

### Quantitative Real-Time Polymerase Chain Reaction (qRT-PCR)

Total RNA was isolated from cells using TRIzol reagent (Thermo Fisher Scientific, Inc.) and then reversely transcripted into cDNA using PrimeScript Reverse Transcriptase (Takara, Japan) following the manufacturer’s instructions. The expression level of gene was measured using the SYBR-Green method, and products were detected by StepOnePlus™ Real-time PCR system (Applied Biosystems, USA). The primers used were as follow:

AKR1B10 (forward), 5'-GACCCCTTGTGAGGAAAGCC-3', AKR1B10 (reverse), 5'- ATTGCAACACGTTACAGGCCC-3';

HOXA5 (forward), 5'-ATCCAAATGGCCCGGACTAC-3', HOXA5 (reverse) 5'- AGTCCCTGAATTGCTCGCTC-3';

GAPDH (forward) 5'-CAACAGCCTCAAGATCATCAGC- 3', GAPDH (reverse) 5'-TTCTAGACGGCAGGTCAGGTC-3'. GAPDH was used as reference gene, and the relative gene expression levels were calculated using the 2^−ΔΔCT^ analysis method.

### Tunel staining

Tunel staining was utilized to observe cell apoptosis through a Tunel Assay kit (Beyotime). Briefly, after being fixed with 4% paraformaldehyde for 30 min, cells were treated with 0.3% Triton X-100 at room temperature for 5 min. Thereafter, 50 μL Tunel detection solution was added to cells and incubated at 37°C for 1 h in the dark. Apoptotic cells were observed under a fluorescence microscope (Olympus Corporation, ×200) after mounting with an anti-fluorescence quenching mounting solution.

### Dual-luciferase report

To verify interactions between HOXA5 and AKR1B10 promoter, wide type (WT) or mutant (MUT) AKR1B10 were cloned into the pGL4 luciferase reporter vectors, and then co-transfected with pcDNA3.1-HOXA5 (Oe-HOXA5) or NC (Oe-NC). At 48 h post-transfection, dual Luciferase Assay (Promega, USA) was applied to determine the luciferase reporter activities according to the manufacturer’s instructions.

### Chromatin immunoprecipitation (ChIP) assay

The ChIP assay was carried out according to a standard protocol. Briefly, after being crosslinked with 1% formaldehyde for 10 min at 37°C, NCI-H295R cells were subjected to ChIP assay with a High-Sensitivity Kit (Abcam). The antibodies used in this assay included anti-HOXA5 and IgG (negative control). The primer sequences of AKR1B10 used for the qRT-PCR assay were: 5'-GACCCCTTGTGAGGAAAGCC-3' (forward), 5'- ATTGCAACACGTTACAGGCCC-3' (reverse).

### Statistical analysis

All data were expressed as the mean ± standard deviation of from three independent experiments for each technique. Statistical analysis was conducted using GraphPad Prism software (version 6.0; GraphPad Software, Inc.). Differences among two groups were analyzed using Student’s test, among multiple groups were assessed using one-way analysis of variance (ANOVA) followed by Tukey’s test. P < 0.05 was considered statistically significant.

## Results

### AKR1B10 is down-regulated in ACC cell lines and overexpression of it inhibits NCI-H295R cells proliferation

**Figure 1.**
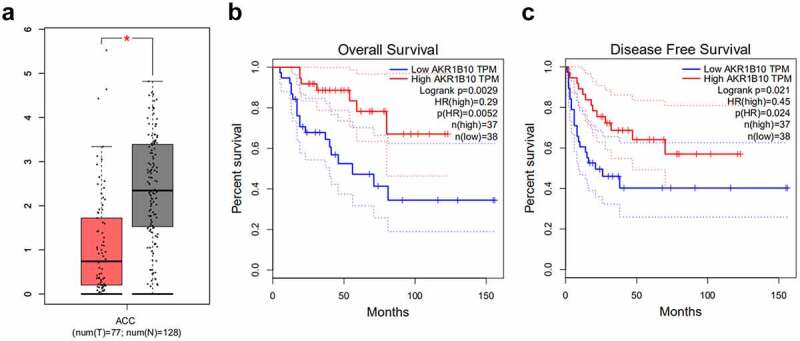
**The relationship between AKR1B10 expression and ACC survival**. A, the expression of AKR1B10 in ACC patients from GEPIA database. The red box represents the ACC group and the gray box represents the normal group. *P < 0.05. B and C, the relationship between AKR1B10 expression and percent survival of OS and DSF of ACC patients from GEPIA database

**Figure 2.**
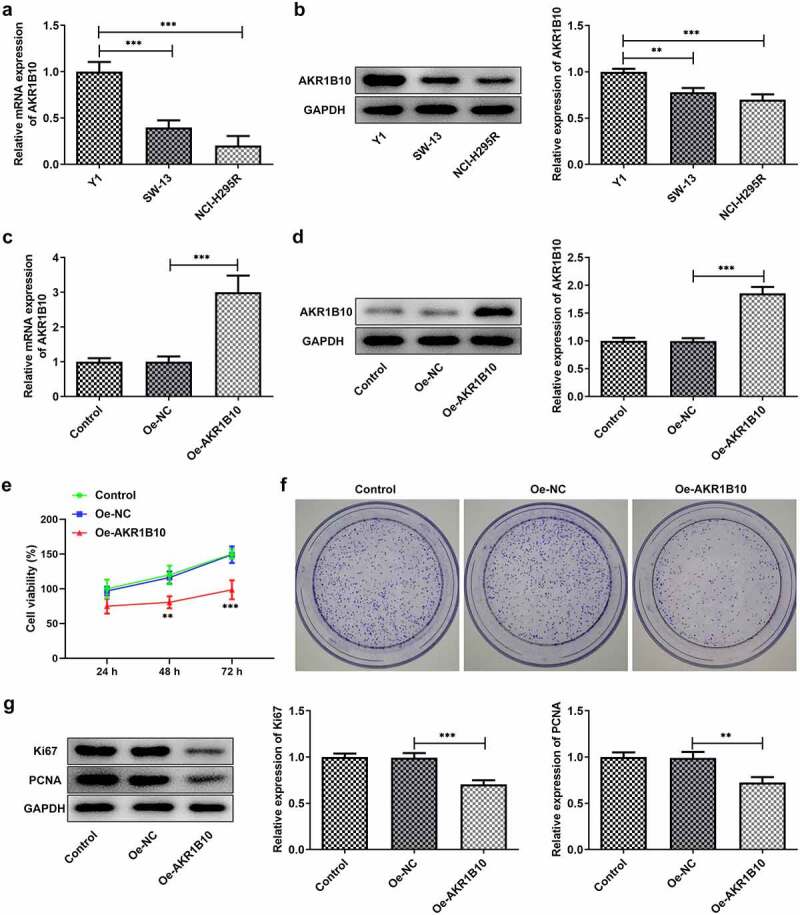
**AKR1B10 overexpression inhibits NCI-H295 cells proliferation**. A and B, the mRNA (a) and protein (b) expressions of AKR1B10 in Y1, SW-13 and NCI-H295 cell lines. C and D, the mRNA (A) and protein (B) expressions of AKR1B10 in NCI-H295 cells before and after AKR1B10 overexpression. E, the cell viability of NCI-H295 cells that overexpressed with AKR1B10 or not at 24, 48 and 72 h. F, the representative images for colony formation assay of NCI-H295 cells that overexpressed with AKR1B10 or not (×100). G, the protein expression of Ki67 and PCNA in NCI-H295 cells that overexpressed with AKR1B10 or not. **P < 0.01 and ***P < 0.001

A considerable body of evidence indicates that AKR1B10 participates in the occurrence and development multiple cancer, such as nasopharyngeal carcinoma, lung cancer and colorectal cancer [7–9]. After searching GEPIA database, we found that AKR1B10 is down-regulated in tissues of ACC patients (Figure 1a). Besides, low level of AKR1B10 is correlated with low overall survival (OS) and disease free survival (DFS), whereas high level of AKR1B10 predicts longer OS and DFS (Figure 1b and c) of ACC, indicating that AKR1B10 plays a beneficial role in inhibiting ACC. Thereafter, we measured the mRNA and protein expression of AKR1B10 in adrenal normal cell line (Y1) and ACC cell lines (SW-13, NCI-H295R). In accordance with the data from GEPIA, AKR1B10 was down-regulated in SW-13 and NCI-H295R cell lines, especially in NCI-H295R, compared with that in Y1 (Figure 2a and b). We then overexpressed AKR1B10 in NCI-H295R cells and results in Figure 2c and d demonstrated the significant increase in AKR1B10 expression. As shown in Figure 2e, AKR1B10 overexpression markedly reduced cell viability at 48 and 72 h. In addition, the colony formation of NCI-H295R cells was markedly inhibited upon AKR1B10 overexpression (figure 2f). Consistently, the expression of proteins involved in cell proliferation including Ki67 and PCNA was also reduced by AKR1B10 overexpression (Figure 2g).

### Overexpression of AKR1B10 induces apoptosis and p53 pathway activation of NCI-H295R cells

**Figure 3.**
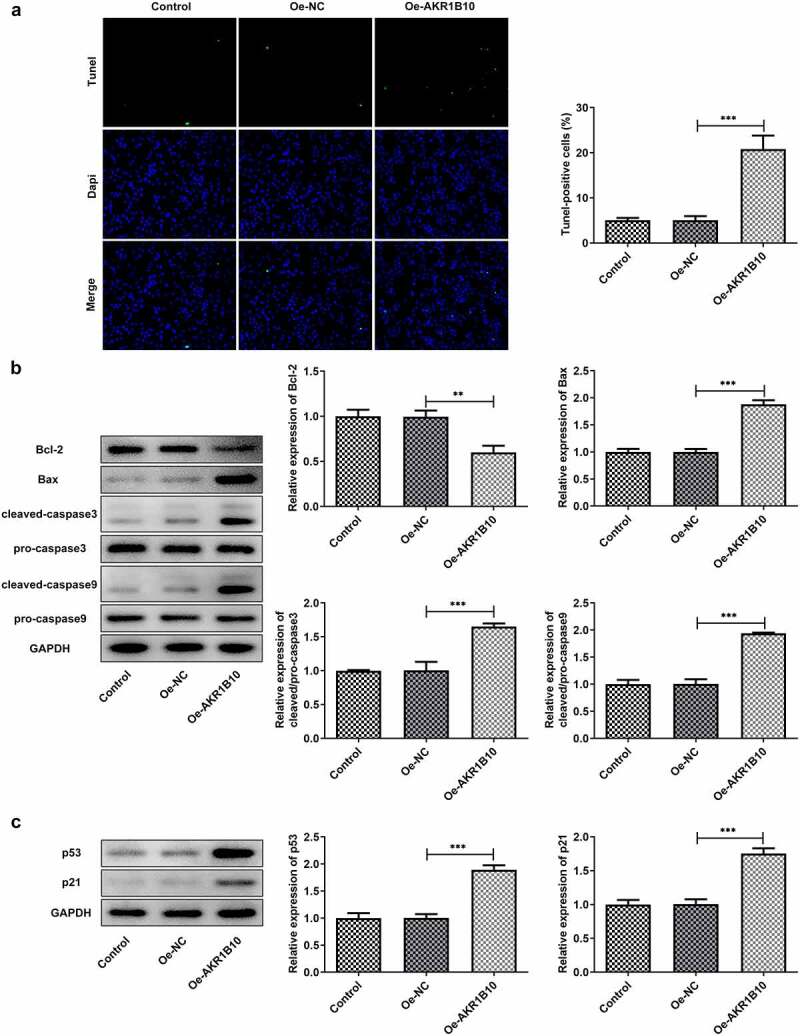
**AKR1B10 overexpression promotes NCI-H295 cells apoptosis and p53 signaling activation**. A, representative images and quantitative analysis for Tunel staining (×200) of NCI-H295 cells that overexpressed with AKR1B10 or not. B, the protein expression of Bcl-2, Bax, c-caspase3 and c-caspase9 in NCI-H295 cells that overexpressed with AKR1B10 or not. C, the protein expression of p53 and p21 in NCI-H295 cells that overexpressed with AKR1B10 or not. **P < 0.01 and ***P < 0.001

To study the effects of AKR1B10-upregulation on the apoptosis of ACC cells, the apoptosis of NCI-H295R cells was evaluated by Tunel staining and western blot assays. As demonstrated in Figure 3a, the number of Tunel-positive cells was highly increased after AKR1B10 overexpression, suggesting the occurrence of apoptosis. At the same time, Bcl-2 expression was reduced, whereas Bax, c-caspase3 and c-caspase9 expressions were remarkably enhanced upon AKR1B10 overexpression, indicating the promotion of AKR1B10 on cell apoptosis (Figure 3b). Besides, AKR1B10 overexpression significantly up-regulated p53 and p51 expressions, revealing the activation of p53 signaling induced by AKR1B10 (Figure 3c).

### HOXA5 is down-regulated in ACC cell lines and can bind to AKR1B10 to regulate AKR1B10 expression

**Figure 4.**
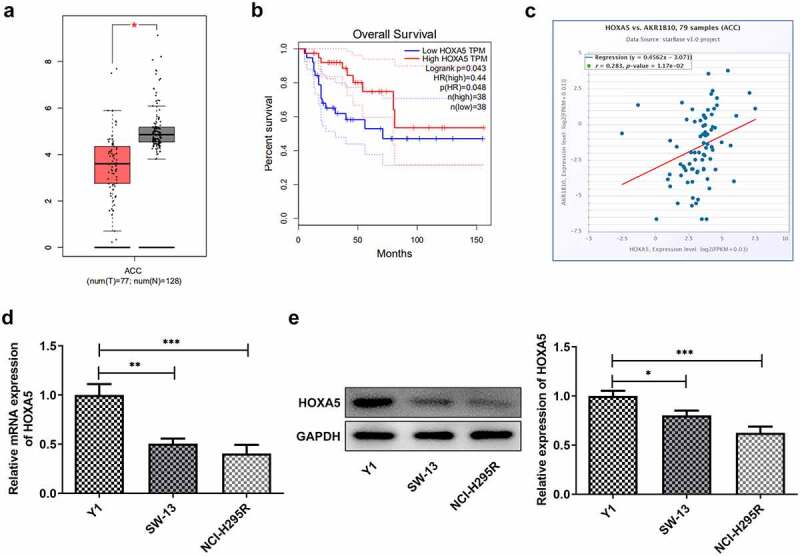
**HOXA5 is down-regulated in ACC cell lines**. A, the expression of HOXA5 in ACC patients from GEPIA database. The red box represents the ACC group and the gray box represents the normal group. *P < 0.05. B, the relationship between HOXA5 expression and percent survival of OS of ACC patients from GEPIA database. C, the relationship between the expression level of AKR1B10 and HOXA5 in ACC samples, data are from ENCORI. D and E, the mRNA (d) and protein (e) expressions of HOXA5 in Y1, SW-13 and NCI-H295 cell lines. *P < 0.05, **P < 0.01 and ***P < 0.001

**Figure 5.**
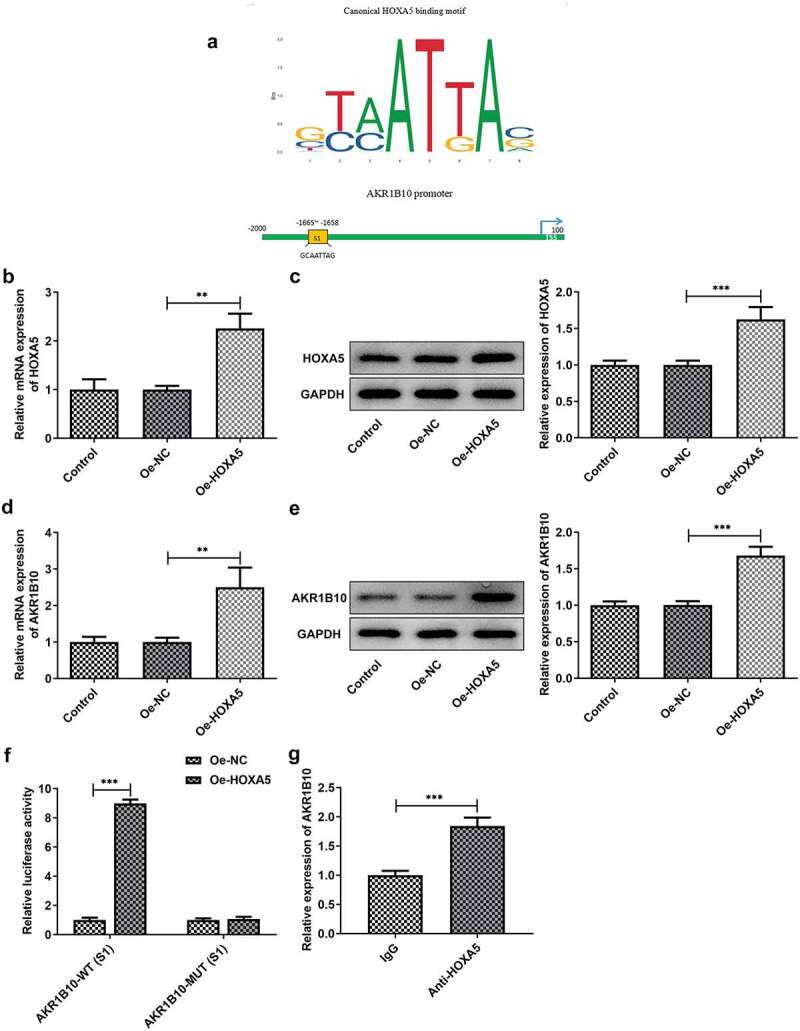
**HOXA5 can bind to AKR1B10 and regulate its expression**. A, the binding sequence between HOXA5 and AKR1B10 promoter predicted by JASPAR website. B and C, the mRNA (b) and protein (c) expressions of HOXA5 in NCI-H295 cells before and after HOXA5 overexpression. D and E, the mRNA (d) and protein (e) expressions of AKR1B10 in NCI-H295 cells before and after HOXA5 overexpression. F and G, the interaction between HOXA5 and AKR1B10 was assessed by dual-luciferase report (f) and ChIP (g) assays. **P < 0.01 and ***P < 0.001

To explore the possible regulatory mechanisms of AKR1B10 in the proliferation and apoptosis of ACC cells, the GEPIA database was employed to assess the expression of HOXA5 in adrenocortical tissues patients with ACC. After searching GEPIA database, we found that HOXA5 is notably down-regulated in ACC tissues (Figure 4a). Meanwhile, low level of HOXA5 is correlated with low OS, whereas high level of HOXA5 predicts longer OS (Figure 4b) of ACC, suggesting that HOXA5 may also play a beneficial role in inhibiting ACC. In addition, data from ENCORI reveals that the expression of HOXA5 and AKR1B10 is positively correlated in ACC (Figure 4c). We then measured the expression of HOXA5 in Y1, SW-13 and NCI-H295R cell lines. As shown in Figure 4d and 4e, HOXA5 was also down-regulated in ACC cells lines, especially in NCI-H295R cell line. The binding sequence between HOXA5 and AKR1B10 promoter was predicted by JASPAR website (Figure 5a). Next, we overexpressed HOXA5 in NCI-H295R cells (Figure 5b and c) to explore the alteration of AKR1B10 expression. Results showed that AKR1B10 expression was remarkably elevated upon HOXA5 overexpression (Figure 5d and e). Moreover, the interaction between HOXA5 and AKR1B10 was validated by dual-luciferase report and ChIP assays (figure 5f and g).These results demonstrated that HOXA5 could bind to AKR1B10 and regulate AKR1B10 expression in ACC cells.

### Knockdown of HOXA5 partially cancels the effects of AKR1B10 overexpression on NCI-H295R cells proliferation and apoptosis

**Figure 6.**
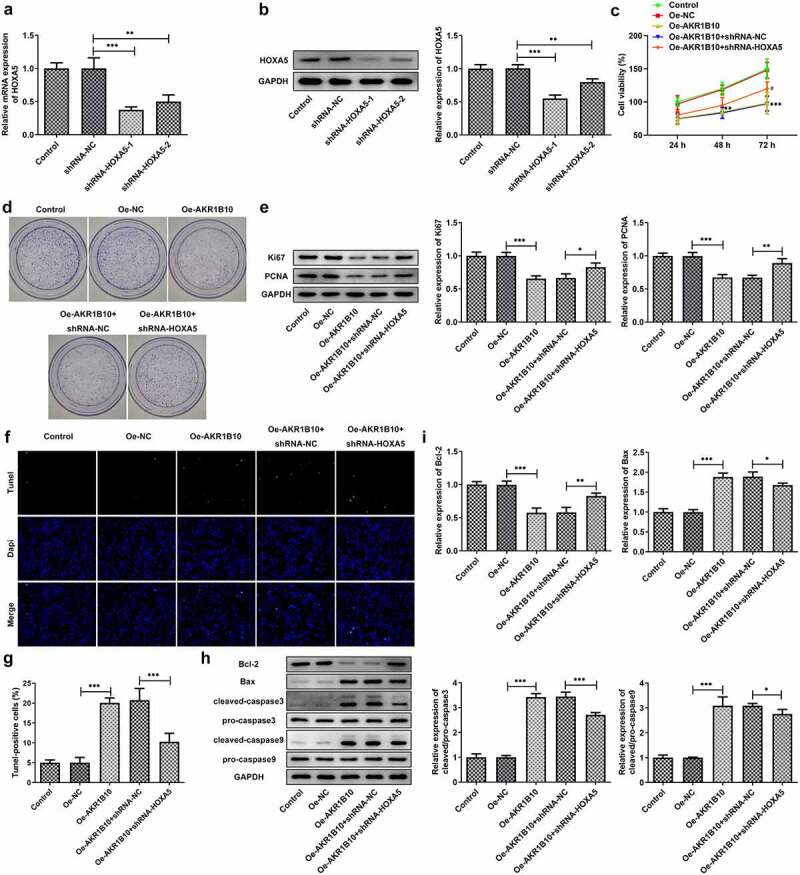
**HOXA5 silence blocks the effects of AKR1B10 overexpression on NCI-H295 cells proliferation and apoptosis**. A and B, the mRNA (a) and protein (b) expressions of HOXA5 in NCI-H295 cells before and after HOXA5 knockdown. C, the cell viability of NCI-H295 cells at 24, 48 and 72 h post-culture. D, the representative images for colony formation assay of NCI-H295 cells in different groups (×100). E, the protein expression of Ki67 and PCNA in NCI-H295 cells in different groups. F and G, representative images and quantitative analysis for Tunel staining (×200) of NCI-H295 cells. H and I, the protein expression of Bcl-2, Bax, c-caspase3 and c-caspase9 in NCI-H295 cells. *P < 0.05

Finally, to investigate whether HOXA5 could regulate the proliferation and apoptosis of NCI-H295 cells via targeting AKR1B10, we knockdown HOXA5 expression in NCI-H295 cells, and chose the shRNA-HOXA5-1 for the following knockdown experiments owing to its better transfection efficacy (Figure 6a and b). NCI-H295 cells that overexpressed with AKR1B10 were silenced with HOXA5 or not, then the proliferation and apoptosis of cells were evaluated. As illustrated in Figure 6c, the decreased cell viability caused by AKR1B10 overexpression was significantly enhanced after HOXA5 silence at 72 h post-culture. What’s more, cells overexpressed with AKR1B10 exhibited an obvious fewer colonies compared with normal cells, however, cells that simultaneously silenced HOXA5 exerted markedly increased number of colonies when compared with cells that only overexpressed with AKR1B10 (Figure 6d). The similar results were observed in Figure 6e, which showed that the silence of HOXA5 increased the expression of Ki67 and PCNA in AKR1B10-overexpressing NCI-H295 cells. figure 6f and g revealed that AKR1B10 overexpression markedly increased the number of apoptotic (Tunel-positive) cells, but HOXA5 knockdown significantly blocked this effect. Consistently, the effect of AKR1B10 overexpression on the expression of proteins related to apoptosis including Bcl-2, Bax, c-caspase3 and c-caspase9, was also partially canceled upon HOXA5 silence (Figure 6h and i).

## Discussion

In the present study, we demonstrated that HOXA5 and AKR1B10 were down-regulated in ACC cells and that the overexpression of AKR1B10 contributed to reduced proliferation and increased apoptosis of ACC cells, but under-expression of HOXA5 was associated with the progression of ACC. In addition, we also demonstrated that HOXA5 could bind to AKR1B10 and enhance its expression in ACC cells. Collectively, we revealed that HOXA5 may exert its anticancer effects through targeting AKR1B10, thereby leading to the inhibition of the abnormal proliferation and induction of apoptosis via activating p53 signaling in ACC cells.

Primary aldosteronism (PA) is one of the common causes of secondary hypertension and can affect up to 13% of hypertensive patients [15]. It has been reported that PA is very likely to cause more serious target organ damage, including cardiovascular and kidney damage, fibrosis, hypertrophy and vascular inflammation [16–18]. In addition to high risk and high prevalence, PA is also characterized by hyper-secretion of aldosterone and adrenal hyperplasia, which is due to hyper-aldosterone secreted by adrenal cells and high proliferation of adrenal cells. Most patients with PA have a benign adrenal adenoma or bilateral hyperplasia [19,20]. ACC, however, is a very rare cause of increased aldosterone levels. The underlying genetic mechanism that leads to ACC and tumor formation is still not fully elucidated.

The data from GEPIA database indicates that the expression of AKR1B10 and HOXA5 is significantly down-regulated in the tissues of patients with ACC, and is related to the poor survival of patients. Our results showed that AKR1B10 and HOXA5 were also down-regulated in ACC cell lines, indicating that they may play an important role in the occurrence and progression of ACC. We then overexpressed AKR1B10 in NCI-H295R cells to observe the change of cell proliferation and apoptosis. We found that AKR1B10 overexpression was able to inhibit proliferation and promote apoptosis of cells, suggesting the inhibitory effect of AKR1B10 on ACC progression. In addition, the p53 signaling was also activated upon AKR1B10 overexpression. p53 and p21 are tumor suppressor proteins that can induce cell cycle arrest and apoptosis of cancer cells [21]. A previous study reported that knockdown of AKR1B10 can significantly inhibit p53-induced apoptosis of CRC cells, while overexpression of AKR1B10 enhances p53-induced apoptosis and inhibits tumor proliferation in vivo [22]. Our data revealed that AKR1B10 may serve as a tumor inhibitor in ACC via activating p53 signaling, thereby inhibiting proliferation and inducing apoptosis of ACC cells.

In CRC, HOXA5 is also down-regulated and its up-expression induces loss of the cancer stem cell phenotype, preventing tumor progression and metastasis [12]. Moreover, HOXA5 has also been reported to cooperate with p53 to play a role in lung cancer mammary tumorigenesis [13,14]. Notably, the data from ENCORI reveals that the expression of HOXA5 and AKR1B10 is positively correlated in ACC, and HOXA5 is predicted to bind to the AKR1B10 promoter after searching JASPAR website. Our results demonstrated that HOXA5 overexpression could increase AKR1B10 expression and the interaction between them was confirmed. Therefore, we speculated that HOXA5 may also function as a tumor suppressor in ACC via targeting AKR1B10 and enhancing its expression level. We subsequently knockdown HOXA5 in the presence of AKR1B10 overexpression to investigate whether the effect of AKR1B10 on cell proliferation and apoptosis could be affected. In accordance with our hypothesis, silence of HOXA5 remarkably abolished the inhibitory effect of AKR1B10 on cell proliferation and the promoting effect of AKR1B10 on cell apoptosis. However, the effect of AKR1B10 on cell proliferation and apoptosis has not been totally reversed by HOXA5 silence. This may be explained by that the expression of HOXA5 has not been fully silenced or maybe there are other proteins and pathways that regulate AKR1B10, which need to be further elucidated in the following research. Additionally, our research is only performed on in vitro cell model, lacking the validation of in vivo evidence. In the subsequent experiments, we will intend to repeat our experiments in animal models to make our conclusions stronger.

### Conclusion

Taken together, our results revealed that HOXA5 could target AKR1B10 to increase AKR1B10 level and activate p53 signaling, thereby inhibiting proliferation and inducing apoptosis of ACC cells, ultimately contributing to the suppression of ACC. Our findings suggested that the approaches that targeting HOXA5/AKR1B10 axis may be a promising therapy in treating ACC.

## Supplementary Material

Supplemental Material

## Highlights

1. AKR1B10 and HOXA5 expression is significantly downregulated in ACC tissues and cells.

2. AKR1B10 overexpression inhibits proliferation and promotes apoptosis of ACC cells.

3. HOXA5 can bind to AKR1B10 promoter to regulate AKR1B10 expression in ACC cells.

4. HOXA5 activates p53 pathway to inhibit ACC progression by binding to AKR1B10.
