# Supplementary figures and images for: Homeobox A5 activates p53 pathway to inhibit proliferation and promote apoptosis of adrenocortical carcinoma cells by inducing Aldo-Keto reductase family 1 member B10 expression
Source: Bioengineered. 2021 May 23;12(1):1964–75. doi: 10.1080/21655979.2021.1924545 (PMC8806264; doi:10.1080/21655979.2021.1924545)

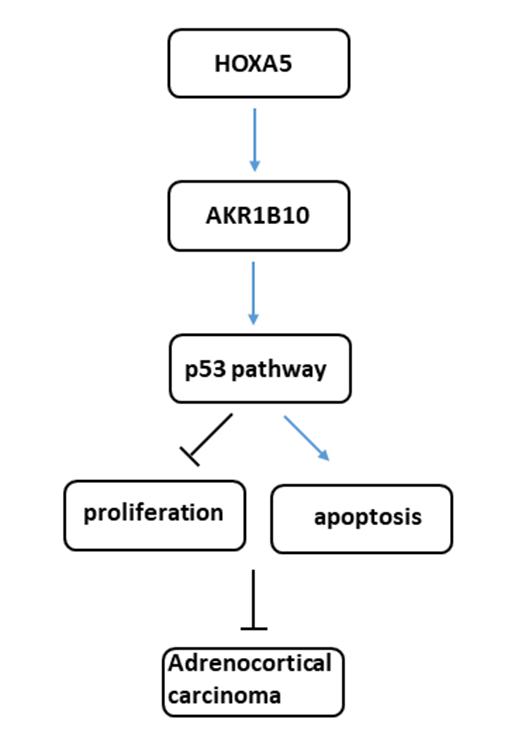

Supplement: Supplemental Material [file KBIE_A_1924545_SM0083.tif]
